# Coherent Raman Scattering Microscopy in Oncology Pharmacokinetic Research

**DOI:** 10.3389/fphar.2021.630167

**Published:** 2021-02-03

**Authors:** Junjie Zeng, Wenying Zhao, Shuhua Yue

**Affiliations:** ^1^ Institute of Medical Photonics, Beijing Advanced Innovation Center for Biomedical Engineering, School of Biological Science and Medical Engineering, Beihang University, Beijing, China; ^2^ Wuhan National Laboratory for Optoelectronics, Huazhong University of Science and Technology, Wuhan, China

**Keywords:** coherent Raman scattering microscopy, label-free, Raman tag, pharmacology, anti-cancer drug, drug metabolism, drug dissolution, drug distribution

## Abstract

The high attrition rates of anti-cancer drugs during clinical development remains a bottleneck problem in pharmaceutical industry. This is partially due to the lack of quantitative, selective, and rapid readouts of anti-cancer drug activity *in situ* with high resolution. Although fluorescence microscopy has been commonly used in oncology pharmacological research, fluorescent labels are often too large in size for small drug molecules, and thus may disturb the function or metabolism of these molecules. Such challenge can be overcome by coherent Raman scattering microscopy, which is capable of chemically selective, highly sensitive, high spatial resolution, and high-speed imaging, without the need of any labeling. Coherent Raman scattering microscopy has tremendously improved the understanding of pharmaceutical materials in the solid state, pharmacokinetics of anti-cancer drugs and nanocarriers *in vitro* and *in vivo*. This review focuses on the latest applications of coherent Raman scattering microscopy as a new emerging platform to facilitate oncology pharmacokinetic research.

## Introduction

Although tremendous efforts have been made to promote anti-cancer drug development (Falzone et al., 2018) (Nass et al., 2018), the high attrition rates of anti-cancer drugs during clinical development remains a bottleneck problem in pharmaceutical industry (Liu et al., 2017). Over the past decade, only 5% of clinically tested anti-cancer drugs have successfully obtained FDA approval (Hay et al., 2014). Considering that anti-cancer drug development is an expensive, time-consuming, and high-risk endeavor, it is necessary to develop novel strategies to identify promising drug candidates and remove ill-fated compounds earlier in the development pipeline.

Imaging has been widely used in anti-cancer drug assessment (Mouras et al., 2010). Due to cell heterogeneity in complex tumor microenvironment, it has become increasingly important to achieve quantitative, selective, and rapid imaging of anti-cancer drug activity *in situ* with high resolution (Vinegoni et al., 2015). Fluorescence microscopy has helped direct visualization of fluorescently labeled molecules, including proteins, antibodies and small molecules such as drugs or their metabolites. Intravital fluorescence microscopy was developed to study anti-cancer drug action *in vivo* at the single-cell level (Thurber et al., 2013), which has been extensively reviewed by Weissleder (Miller and Weissleder 2017).

Although fluorescence microscopy has been commonly used in oncology pharmacological research, fluorescent labels are often large in size relative to small drug molecules, and thus may perturb the activity of these drug molecules. Thus, label-free optical microscopy that generates signals based on intrinsic molecular contrast would be desirable to study uptake, distribution, and metabolism of small drug molecules.

The efficacy of anti-cancer drugs also largely relies on drug delivery vehicles (e.g., tablets and nanocarriers) (Shi et al., 2017), which are formulated to enhance drug bioavailability, biocompatibility, and targeting to cancer tissues. Thorough understanding of the drug stability and activity within the final dosage form is required to optimize the dosing strategy and reduce toxic effects prior to regulatory approval. Encapsulation of fluorescent dyes has been commonly used to monitor nanocarriers, but this is limited by occasional loss and photobleaching of the dyes. Thus, it would be of great importance to develop label-free optical microscopy that can assess stability and dissolution of drugs in the solid state, and uptake, distribution, interaction, and excretion of anti-cancer drug nanocarriers in a biological environment.

Based on intrinsic contrasts from molecular vibrations, infrared absorption and Raman scattering offer attractive means for label-free chemical-selective imaging. Compared to infrared absorption, Raman scattering based imaging would have higher spatial resolution by use of visible/near-infrared light excitation. Moreover, different from infrared absorption, Raman scattering does not have background from water, which makes it much more suitable to study live biological systems. Despite that spontaneous Raman microscopy has been used in oncology pharmacokinetic research (Gala and Chauhan 2015), small cross section of Raman scattering makes it difficult to acquire strong enough signals for fast chemical imaging, which significantly hinders its application in dynamic readouts of anti-cancer drug activity *in situ*.

**FIGURE 1:**
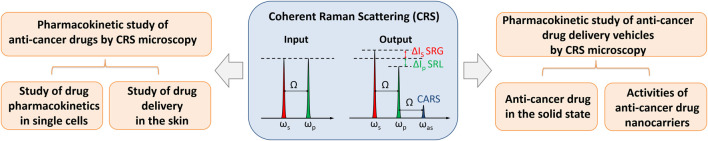
Schematic showing coherent Raman scattering (CRS) microscopy and its application in oncology pharmacokinetics. When tuning the beating frequency (*ω*_p_-*ω*_s_) to match molecular vibration frequency *Ω*, multiple Raman transitions were excited simultaneously, including stimulated Raman gain (SRG), stimulated Raman loss (SRL), and coherent anti-Stokes Raman scattering (CARS). *ω*_p_, *ω*_s_, and *ω*_as_ denote the frequencies of the pump, Stokes, and anti-Stokes beam, respectively. CRS microscopy has shown great potential in the investigation of anti-cancer drug pharmacokinetics, anti-cancer drug stability and dissolution in the solid state, and activities of anti-cancer drug nanocarriers in single cells.

In order to enhance the Raman scattering signal, coherent Raman scattering (CRS) microscopy has been developed (Cheng and Xie 2012). When tuning the beating frequency to match a molecular vibration frequency, the CRS signal can be markedly boosted and so enables high-speed imaging, which is 10^4^–10^6^ times faster than spontaneous Raman microscopy (Cheng and Xie 2015) (Figure 1). Raman tags with distinct Raman peaks in cellular “silent region” have been shown to enhance molecular selectivity of CRS microscopy without perturbing biological activities (Wei et al., 2016). Owing to these unique advantages, CRS microscopy offer a powerful platform to study anti-cancer drug stability and activity within the final dosage form.

Here, we review the recent technical advances and applications of CRS microscopy in the study of anti-cancer drug pharmacokinetics at the single cell level, drug stability and dissolution in the solid state, and activities of anti-cancer drug nanocarriers in single cells (Figure 1). We then conclude with the discussion on the potential of CRS microscopy to promote oncology pharmacokinetic research.

## CRS Microscopy

In CRS microscopy, two ultra-short pulse excitation beams are used, denoted as pump (*ω*_p_) and Stokes (*ω*_s_) respectively. When the beating frequency (*ω*_p_ − *ω*_s_) is in resonant with a molecular vibration frequency (*Ω*), the Raman scattering signal can be dramatically increased in coherent anti-Stokes Raman scattering (CARS) and stimulated Raman scattering (SRS) processes (Cheng and Xie 2012). The advantages of SRS over CARS lie in the fact that the SRS signal is completely free of non-resonant background, which makes SRS microscopy a highly sensitive method for biochemical imaging (Freudiger et al., 2008). In an effort to gain spectral information, hyperspectral CRS microscopy has been developed based on frame-by-frame wavelength scanning, which may take seconds to minutes to obtain an entire stack of images for reconstruction and leads to some spectral distortions. To avoid such distortion, multiplex CRS microscopy, where a CRS spectrum is instantaneously recorded at each pixel (microseconds per pixel), has been developed. These instrumental advancements and accompanied improvements in chemical map decomposing algorithms have been extensively reviewed in (Zhang et al., 2014; Cheng and Xie 2015; Fu 2017). Owing to the fast, label-free, and chemical-selective imaging capability, CRS microscopy has been widely used in biomedical research, as reviewed in (Min et al., 2011; Pezacki et al., 2011; Streets et al., 2014; Camp and Cicerone 2015; Schie et al., 2015; Winterhalder and Zumbusch 2015; Zhang et al., 2015; Yue and Cheng 2016; Zhang and Cheng 2018; Hill and Fu 2019; Hu et al., 2019).

The molecular selectivity and detection sensitivity of CRS microscopy can be further enhanced by small-sized Raman tags (e.g., deuterium, alkyne, and diyne), which show strong Raman peaks well separated from endogenous cellular signals without perturbing biological activities of small molecules. Based on this method, cellular uptake, distribution, and metabolism of small molecules can be monitored with high temporospatial resolution and high detection sensitivity (micromolar level) *in vitro* and *in vivo*, as reviewed in (Wei et al., 2016; Hu et al., 2019).

## Pharmacokinetic Study of Anti-Cancer Drugs by CRS Microscopy

Raman spectroscopy and microscopy can be used to investigate pharmacokinetics in living cells with high resolution and in a label-free manner. El-Mashtoly and co-workers have made tremendous efforts to push the study of pharmacokinetics via label-free molecular fingerprint (El-Mashtoly et al., 2014; El-Mashtoly et al., 2015; Aljakouch et al., 2018; Yosef et al., 2018; El-Mashtoly 2020). More recently, CRS microscopy has been increasingly employed for high-speed imaging of the uptake, distribution, and metabolism of anti-cancer drugs in single live cells *in vitro* and *in vivo* (Tipping et al., 2016). As discussed below, these studies show great impact on mechanistic understanding of the anti-cancer drug activity and may significantly accelerate the preclinical medicinal chemistry optimization pipelines.

### Study of Drug Pharmacokinetics in Single Cells by CRS Microscopy

**FIGURE 2:**
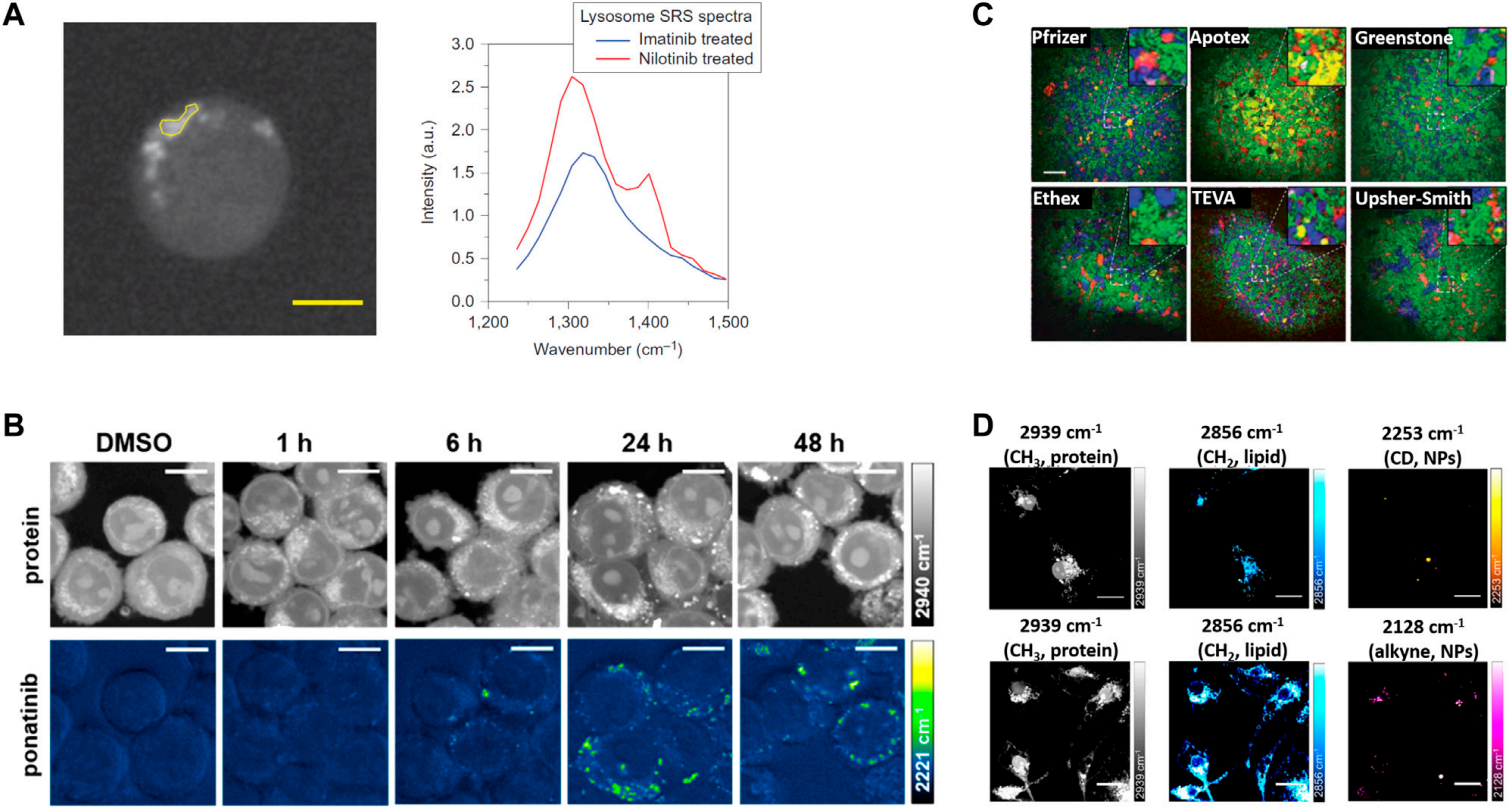
The representative applications of coherent Raman scattering (CRS) microscopy in oncology pharmacokinetics. **(A)** Representative SRS images at 1305 cm^−1^ of BaF3/BCR-ABL1 cells treated with 20 mM nilotinib for 4 h. SRS spectra of selected ROI in the left image (yellow polygon). Reprinted with permission from Ref. (Fu et al., 2014). Copyright 2014 Nature Publishing Group. **(B)** SRS imaging of ponatinib uptake in KCL22^Pon−Res^ cells. KCL22^Pon−Res^ cells were treated with DMSO (0.0003%, v/v) or ponatinib (500 nM) for 1, 6, 24, or 48 h. SRS images acquired at 2940 cm^−1^ (CH_3_, proteins), 2221 cm^−1^ (C≡C, ponatinib), Scale bars: 10 μm. Reprinted with permission from Ref. (Sepp et al., 2020). Copyright 2019 American Chemical Society. **(C)** Large area SRS imaging of tablets. Green, blue, red, yellow/orange, and magenta colors represent microcrystalline cellulose, dibasic calcium phosphate anhydrous, Amlodipine besylate (API), sodium starch glycolate, and magnesium stearate, respectively. In case of tablet from Apotex the yellow color corresponds to lactose monohydrate and corn starch. Inserts are four times magnified areas of the images indicated by dashed squares. Scale bar: 200 μm. Reprinted with permission from Ref. (Slipchenko et al., 2010). Copyright 2021 Royal Society of Chemistry **(D)** SRS imaging of nanoparticles (NPs) in microglia. Microglia were incubated with PLGA-CD NPs or PLGA-alkyne NPs. Scale bars: 20 μm. Reprinted with permission from Ref. (Vanden-Hehir et al., 2019a). Copyright ^©^ 2019 American Chemical Society.

In 2014, Xie group, for the first time, demonstrated that hyperspectral SRS microscopy enabled label-free visualization and quantification of tyrosine-kinase inhibitors (imatinib and nilotinib) (Figure 2A), which are the front-line therapies for chronic myelogenous leukemia, inside living cells (Fu et al., 2014). Both tyrosine-kinase inhibitors were shown to enrich over 1,000-fold in lysosomes, probably due to low solubility. Moreover, this work unraveled a new mechanism by which chloroquine could increase the efficacy of tyrosine-kinase inhibitors, that is, lysosome-mediated drug-drug interaction.

Recently, Raman tagging strategies have been shown to enhance early stage drug discovery programmes. In 2014, Min group, for the first time, imaged the delivery pathways of alkyne-bearing terbinafine hydrochloride, a US Food and Drug Administration-approved antifungal drug, inside mouse ear skin tissue (Wei et al., 2014). Subsequently, Min group and collaborators used a Raman tag to determine the subcellular localization and mechanism of action of ferrostatins in suppressing ferroptosis, a form of nonapoptotic cell death (Gaschler et al., 2018). Min and coworkers further employed a Raman tag to study the intracellular enrichment and distribution of the anti-cancer antimycin-type depsipeptides in single live cells (Seidel et al., 2019). Hulme group demonstrated the feasibility of SRS microscopy to evaluate subcellular distribution of Phenyl-based Raman tag labeled anisomycin, and cellular response to the drug simultaneously (Tipping et al., 2017). Huang group reported hyperspectral SRS imaging of Diyne-based Raman tag labeled Triphenylphosphonium, a commonly used mitochondria-targeting agent, to track the dynamics of mitochondria in live cells (Bae et al., 2020). More recently, Hulme group took advantages of alkyne-based SRS imaging to assess label-free uptake and distribution of ponatinib, another tyrosine kinase inhibitor approved for chronic myeloid leukemia treatment, in cellular models of ponatinib resistance (Sepp et al., 2020) (Figure 2B). This study achieved at biologically relevant, nanomolar concentrations, allowing determination of changes in uptake and sequestration of ponatinib during the development of acquired drug resistance. Taken together, these works highlight the great potential of bioorthogonal chemical imaging (CRS microscopy of Raman tags) in anti-cancer drug pharmacokinetics research.

### Study of Drug Delivery in the Skin by CRS Microscopy

The effective treatment of diseases of the skin remains an important unmet medical need, primarily because of poor drug delivery. To address this challenge, SRS microscopy has been used to visualize and characterize the diffusion of topically applied drugs into mammalian skins in real time. Ten years ago, Xie group developed video-rate SRS microscopy to study the penetration pathways of topically applied drugs in mice and humans non-invasively without any labeling (Saar et al., 2010). Their later study further revealed different rates of drug penetration via hair follicles as compared to the intercellular pathway across the stratum corneum (Saar et al., 2011). The high-speed three-dimensional imaging capability allowed SRS microscopy to provide mechanistic insight into the (trans)dermal drug delivery process. Similarly, Guy group studied diffusion of deuterated water, propylene glycol, and dimethyl sulfoxide in the human nail by SRS imaging of the O-D, -CD_2_, and -CH_2_ bond, respectively (Chiu et al., 2015). Taking advantage of alkyne-bearing drug, Min group imaged the delivery pathways of terbinafine hydrochloride, an antifungal skin drug, inside mouse ear tissue to a depth of about 100 μm (Wei et al., 2014). More recently, Evans group made use of deep learning-based computational methods to analyze SRS images, which help to quantify the flow and flux of small molecule drugs through the layers and structures of nude mouse ear skin (Feizpour et al., 2020; Pena et al., 2020). Although current *in vivo* pharmacokinetic studies by CRS microscopy are not directly related to anti-cancer treatment, the strategies demonstrated in these studies may open a new avenue for skin cancer pharmacokinetics *in vivo*.

Taken together, CRS microscopy provides unprecedented capabilities in dynamic visualization of drug activity in single cells and offer new insight into drug discovery and development.

## Pharmacokinetic Study of Anti-Cancer Drug Delivery Vehicles by CRS Microscopy

Modern regulations demand thorough knowledge of drug stability and activity within the final dosage form (Yu et al., 2014). Thus, it is essential to assess drug delivery vehicles (e.g., tablets and nanocarrier) that are made to ensure the prolonged stability and bioavailability of the drug. The non-destructive and label-free features of Raman and infrared imaging make them desirable analytical tools to assess drug delivery vehicles, as reviewed in (Gowen et al., 2008; Gordon and McGoverin 2011). Owing to the advantage of high-speed imaging compared to spontaneous Raman microscopy, CRS microscopy has been increasingly used for pharmacokinetic study of anti-cancer drug delivery vehicles, in order to meet the quality by design demands in the modern pharmaceutical industry.

### Anti-Cancer Drug Stability and Dissolution in the Solid State

Pharmaceutical tablets are composed of biologically active pharmaceutical ingredients (API) and inert excipients that ensure the pharmacological properties of the API. The therapeutic performance and stability of the final dosage form are affected by the spatial distribution and phase of API and excipients. Due to heterogeneous nature of solid drug formulations, it is essential to characterize the distribution and dissolution of drug formulations noninvasively at high temporospatial resolution. Below we will review the applications of CRS microscopy in assessment of API crystallization and polymorphism, chemical mapping of final dosage forms, and *in-situ* chemical imaging during dissolution.

Polymorphism refers to the ability of a molecule to crystallize into multiple crystal structures. In the development of an API, it is critical to identify polymorphic forms to ensure API stability, dissolution kinetics, and bioavailability. Cicerone group, for the first time, used broadband CARS microscopy with much higher speed and spatial resolution compared to spontaneous Raman imaging, allowing identification of three crystalline polymorphs and an unanticipated API phase within a tablet (Hartshorn et al., 2013). Slepkov group implemented spectral-focusing-CARS microscopy, with broadband hyperspectroscopy and rapid single vibrational frequency imaging, to discriminate ibuprofen, common polymorphs of acetaminophen, and starchy binders on tablet samples (Porquez and Slepkov 2018).

Chemical mapping of final dosage forms is essential to validate a uniform distribution of ingredients within the tablet for reliable product performance. In 2010, Cheng group, for the first time, demonstrated SRS microscopy could map API (amlodipine besylate, a widely used drug for lowering the blood pressure) and a variety of excipients, including microcrystalline cellulose, dibasic calcium phosphate anhydrous, sodium starch glycolate, and magnesium stearate, with high chemical selectivity and high temporospatial resolution (Slipchenko et al., 2010) (Figure 2C). More recently, Rigneault group showed that few SRS images at selected wavenumbers could retrieve molecular maps of both API (clopidogrel and amibegron) polymorphs and excipients (polyethylene glycol, corn starch, and mannitol) over millimeter-size areas within compact tablets (Sarri et al., 2019).

Dissolution testing, which monitors API dissolution kinetics under conditions mimicking those *in vivo*, is an indispensable step in drug product development and quality control. As early as 2006, Cheng group and Park group demonstrated CARS microscopy could be used to examine distribution of anti-cancer drug paclitaxel based on its specific Raman peaks in polyethylene glycol (PEG) and poly (lactic-co-glycolic acid) (PLGA) films with high spatial resolution (Kang et al., 2006). This study further monitored the dynamic release of paclitaxel from a polymer matrix during dissolution *in situ*. Strachan group used CARS microscopy to monitor the dissolution of the model drug theophylline in lipid-based oral dosage forms, and found the drug tended to form the less soluble monohydrate during dissolution (Windbergs 2009). Their later study used hyperspectral CARS to monitor the solid-state change in oral dosage forms containing theophylline anhydrate during dissolution and found that theophylline anhydrate converted to theophylline monohydrate resulting in a reduction in the dissolution rate (Fussell et al., 2013).

More recently, SRS microscopy has been utilized for *in situ* chemical imaging of drug release and interaction with formulations. Fu group made use of SRS microscopy for chemical mapping of entecavir, a hepatitis B antiviral drug, embedded in a slow release poly (D,l-lactic acid) formulation. High spatial resolution of SRS microscopy allowed quantitative profiling of dissolution of single crystalline particles in implant formulations *in situ* (Francis et al., 2018). Their later work demonstrated chemical imaging of salt disproportionation reaction of pioglitazone hydrochloride (PIO-HCl) at a very low drug loading (1% w/w) by SRS microscopy (Figueroa et al., 2019). Huang group utilized Raman-tagged hyperspectral SRS microscopy to study dynamic interplay between antibiotics and biofilm, which is crucial for understanding of antibiotics resistance (Bae et al., 2019).

Furthermore, CRS microscopy can be integrated with other nonlinear optical (NLO) microscopy modalities, such as two- and three-photon fluorescence, second- and third-harmonic generation, on the same platform. Such multimodal NLO microscopy combined advantages of each phenomenon for imaging complex pharmaceutical systems. More details can be found in (Strachan et al., 2011; Novakovic et al., 2017; Schmitt 2017; Ojarinta et al., 2018).

### Activities of Anti-Cancer Drug Nanocarriers in Single Cells

Nanocarriers are designed to enhance drug bioavailability, biocompatibility, and targeting to cancer tissues, which may lead to more effective and safer cancer treatment. The field of cancer nanomedicine has gained considerable technological success (Doane and Burda 2012; Wicki et al., 2015; Shi et al., 2017; Begines et al., 2020), but is currently facing several obstacles for clinical translation, especially little knowledge about nano-bio interactions (Shi et al., 2017). Raman microscopy has been utilized for noninvasive imaging of pharmaceutical nanocarriers (Chernenko et al., 2012; Chernenko et al., 2013; Vanden-Hehir et al., 2019b), and high chemical selectivity and high temporospatial resolution make CRS microscopy a more attractive way to study activities of anti-cancer drug nanocarriers in single cells and cellular response to the drugs.

As early as 2009, Yeo and Cheng groups used label-free CARS microscopy to reexamine cellular uptake of poly (lactic-co-glycolic acid) (PLGA) nanoparticles (NPs), and discussed the utility and limitations of PLGA NP as an intracellular drug delivery system (Xu et al., 2009). Garrett et al. presented CARS as a novel tool for label-free imaging of polymeric NPs in biological cells and tissues (Garrett et al., 2012a). Van den Mooter group showed the potential of CARS to investigate drug nano-/microcrystal–cell interactions in cell cultures and *ex vivo* in histological sections without labeling (Darville et al., 2015). Tolstik et al. used CARS for precise detection of the uptake of biodegradable non-toxic silicon NPs by cancer cells (Tolstik et al., 2016).

Recently, Wang group, for the first time, used hyperspectral SRS to investigate the subcellular distribution of NPs in the protozoan *Tetrahymena* thermophila, and found the two frequently studied nanoparticles, polyacrylate-coated alpha-Fe_2_O_3_ and TiO_2_, had significant uptake competition and different subcellular distribution pattern (Huang et al., 2018). Hulme group synthesized alkyne-tagged PLGA to show direct visualization of nanoparticles *in vitro* within primary rat microglia and *ex vivo* cortical mouse brain tissue by SRS microscopy (Vanden-Hehir et al., 2019a) (Figure 2D).

Furthermore, multimodal NLO microscopy, which integrates CRS microscopy, two-photon fluorescence microscopy, and second harmonic generation, has shown great potential in investigation of cellular uptake and tissue distribution of drug nanocarriers. Garrett et al. utilized such label-free multimodal NLO microscopy to pinpoint polymeric NPs within the stomach, intestine, gall bladder and liver (Garrett et al., 2012b). Johnston et al. used CRS-based multimodal NLO microscopy to evaluate nanomaterial-cell interactions by visualizing the uptake of gold or titanium dioxide nanomaterials in live and fixed cell lines and biodistribution of nanomaterials in lung and liver tissues in rats (Johnston et al., 2015).

Taken together, in order to optimize the dosing and reduce unwanted toxic effects, it is advantageous of CRS microscopy in label-free and non-invasive imaging drug nanocarrier activity at the single-cell level, which offers a deeper understanding of how nanocarriers interact with cells and tissues, as also reviewed in (Goodhead et al., 2015; Vanden-Hehir et al., 2019b).

## Concluding Remarks

Given the time and overall costs required to bring novel therapeutics to patients, new technologies capable of providing earlier feedback and deeper understanding during the initial phases of drug development and validation are of great importance and urgent need. Owing to the capability of label-free, chemically selective, high temporospatial resolution, and highly sensitive imaging, CRS microscopy has remarkably improved the understanding of anti-cancer drug pharmacokinetics *in vitro* and *in vivo*. Advances in Raman tag design are expected to significantly enhance the detection sensitivity and selectivity, which may offer new opportunities for investigation of anti-cancer drugs that do not have distinctive Raman peaks. Advances in instrumental development, such as novel hyperspectral and multiplex CRS microscopy, are expected to promote high-speed simultaneous imaging of multiple drug molecules and drug vehicles. Furthermore, integration of multimodal NLO microscopy techniques is expected to shed new light on the understanding of how drug molecules and drug vehicles interact with cells within the complex tumor tissue environment.

## Author Contributions

JZ and WZ contributed to the systematic review of literatures and wrote the initial draft of the section about tablets. JZ also revised the manuscript according to reviewers’ comments. SY wrote the manuscript, critically analyzed and revised the manuscript according to reviewers’ comments.

## Funding

This work is supported by National Natural Science Foundation of China (No. 91959120, No. 62027824 to SY), Beijing Natural Science Foundation (No. L172011 to SY), Open Project Program of Wuhan National Laboratory for Optoelectronics (No. 2018WNLOKF026 to SY), Fundamental Research Funds for the Central Universities (No. YWF-20-BJ-J-550 to SY), and “Excellent Hundred Talents” Program start-up fund from Beihang University (to SY).

## Conflict of Interest

The authors declare that the research was conducted in the absence of any commercial or financial relationships that could be construed as a potential conflict of interest.
